# Maternal thoughts of self-harm and their association with future offspring mental health problems

**DOI:** 10.1016/j.jad.2021.06.058

**Published:** 2021-10-01

**Authors:** Elise Paul, Alex Kwong, Paul Moran, Susan Pawlby, Louise M. Howard, Rebecca M Pearson

**Affiliations:** a Department of Behavioural Science and Health at University College, London, United Kingdom; b Centre for Academic Mental Health, Population Health Sciences, Bristol Medical School, University of Bristol, Oakfield House, Bristol, United Kingdom; c Faculty of Life Sciences & Medicine, School of Life Course Sciences, King's College London, London, United Kingdom; d Section of Women's Mental Health, Institute of Psychiatry, Psychology and Neuroscience, King's College London, London, United Kingdom

**Keywords:** ALSPAC, Longitudinal studies, COVID-19, Child development

## Abstract

**Introduction:**

Depression and self-harm are leading causes of disability in young people, but prospective data on how maternal depression and self-harm thoughts contribute to these outcomes, and how they may interact is lacking.

**Methods:**

The study sample consisted of 8,425 mothers and offspring from the Avon Longitudinal Study of Parents and Children, an ongoing birth cohort study. Exposures were maternal self-harm ideation and depression measured using the Edinburgh Postnatal Depression Scale, collected at eleven time points over the period 18 weeks’ gestation to 18 years post-partum. Outcomes were offspring past-year major depressive disorder and lifetime self-harm assessed at age 24.

**Results:**

Nearly one-fifth (16.7%) of mothers reported thoughts of self-harm on at least one of the eleven assessment points. The frequency of maternal self-harm ideation was related to both outcomes in a dose-response manner. Young adults whose mothers had self-harm ideation on 5–11 occasions were over three times more likely (Odds ratio (OR), 3.32; 95% CI, 1.63–6.76) to be depressed and over 1.5 times as likely (OR, 1.55; 95% CI, 0.73, 3.29) to have self-harmed than their peers whose mothers had never reported self-harm thoughts. Maternal self-harm thoughts remained associated with both offspring outcomes independent of maternal depression, and no evidence was found for an interaction between the two exposures.

**Discussion:**

Clinicians collecting data on maternal depression may consider paying attention to questions about self-harm ideation in assessments. Examining accumulated maternal self-harm ideation over time may provide insights into which children are most at risk for later self-harm and depression.

## Highlights

• Maternal self-harm ideation predicted long-term offspring depression and self-harm.
• This relationship occurs independently of maternal depression.
• Analyses also indicate a dose-response effect.
• No interaction of maternal depression and self-harm thoughts with offspring outcomes.

## Introduction

Depression and self-harm represent a significant public health burden. A diagnosis of depression is amongst the most important risk factors for thoughts of self-harm (Franklin et al., 2017), and both represent increased risk for these and other negative outcomes in children when they occur in mothers (Geulayov et al., 2012, Hammerton et al., 2016; Hammerton et al., 2016; Netsi et al., 2018; Pearson et al., 2013; Stein et al., 2014). Persistent maternal depression is associated with substantially elevated risk for offspring depression (Hammen and Brennan, 2003; Netsi et al., 2018), but less is known about whether the persistence of maternal thoughts of self-harm (versus the occurrence of single episodes) is associated with adverse offspring mental health outcomes.

Maternal thoughts of self-harm are associated with greater severity of depression (Howard et al., 2011) which, in turn, may be associated with greater mental health difficulties in children (Hammen and Brennan, 2003). The impact of timing of maternal depression on offspring outcomes is unclear (Brennan et al., 2000; Hammen and Brennan, 2003). Furthermore, even less is known regarding the timing of maternal self-harm ideation on child mental health outcomes.

In the UK, current NICE (National Institute for Health Care and Excellence) guidelines encourage routine screening for depression in expectant mothers, with the Edinburgh Postnatal Depression Scale (EPDS) (Cox et al., 1987) recommended as a follow up to those who screen positive on either of two depression screening questions in the UK ((National Institute for Health and Care Excellence, 2020)). The EPDS contains an item enquiring about thoughts of self-harm, and mothers endorsing this item experience more severe depressive symptoms (Borschmann et al., 2019). Examining long-term child outcomes of maternal self-harm ideation may therefore help in the early identification of mothers and infants most at risk and guide clinical decision- making about future management.

We extend prior work on maternal self-harm ideation (Howard et al., 2011) and depression (Netsi et al., 2018) by examining prospective associations between frequency and timing of maternal self-harm ideation and depression beyond the perinatal period and offspring mental health, over 11 successive waves of follow-up conducted over a 17-year period. In addition, we set out to determine whether an interaction between maternal thoughts of self-harm and depression conveys greater offspring risk for mental health problems. We hypothesised that the offspring of mothers with both self-harm ideation and depression would be at greater risk for worse mental health outcomes compared with the offspring of mothers with neither compare to just one of these exposures.

## Methods

### Study cohort

The study sample comprised participants from the Avon Longitudinal Study of Parents and Children (ALSPAC). A total of 14,451 pregnant mothers residing in the former Avon Health Authority in the south-west of England with expected dates of delivery between 1 April 1991 and 31 December 1992 were initially enroled in the study. These pregnancies resulted in 14,062 live births, of which 13,998 were alive at 1 year of age. For further details on the cohort profile, representativeness, and phases of recruitment, see Boyd et al. (2013), Fraser et al. (2013) and Northstone et al. (2019). The study website contains details of all data available through a fully searchable data dictionary and variable search tool (http://www.bristol.ac.uk/alspac/researchers/our-data/). Study data were collected and managed using REDCap electronic data capture tools hosted at the University of Bristol (Harris et al., 2019, 2009). REDCap (Research Electronic Data Capture) is a secure, web-based software platform designed to support data capture for research studies. This report follows the Strengthening the Reporting of Observational Studies in Epidemiology (STROBE) reporting guideline (Elm et al., 2007).

### Ascertainment of maternal self-harm ideation and depression

The 10-item Edinburgh Postnatal Depression Scale (EPDS) (Cox et al., 1987) was administered to mothers at 11 time points starting with 18 weeks’ gestation through 18 years post-birth (see Table 1 for complete list of time points). The EPDS assesses depression symptoms over the prior two weeks. Mothers rated the frequency of each EPDS item, including the question on thoughts of self-harm, from (0) *never*, (1) *hardly ever*, (2) *sometimes*, to (3) *quite often*. Maternal self-harm ideation was measured with item 10 of the EPDS: *“The thought of harming myself has occurred to me*.” To maintain consistency with prior work (Gordon et al., 2019a; Howard et al., 2011), we coded responses of *never* and *hardly ever* as having no self-harm ideation and responses of *sometimes* or *quite often* as having self-harm ideation.

**Table 1:** Characteristics of the study sample (*N* = 8425).

Variable	% a / Mean (SE)
Child is female	48.72%
Maternal education	
O-level or lower	59.31%
A-level	25.36%
University degree	15.33%
Maternal marital status	
Married	80.92%
Maternal age in years	28.22 (0.05)
Offspring past-year major depressive disorder, age 24	11.11%
Offspring lifetime self-harm, age 24	19.28%
Maternal self-harm ideation timing b	
18 weeks’ gestation, T1	4.23%
32 weeks’ gestation, T2	2.24%
8 weeks post-partum, T3	1.52%
8 months post-partum, T4	2.24%
1 year 9 months post-partum, T5	2.17%
2 years 9 months post-partum, T6	2.70%
5 years post-partum, T7	2.52%
6 years post-partum, T8	2.16%
8 years post-partum, T9	2.50%
11 years post-partum, T10	1.91%
18 years post-partum, T11	5.40%
Maternal self-harm ideation frequency	
Never	83.33%
1–4 times	15.67%
5–11 times	1.00%
Maternal depression timing c	
18 weeks’ gestation, T1	11.06%
32 weeks’ gestation, T2	13.14%
8 weeks post-partum, T3	8.66%
8 months post-partum, T4	8.02%
1 year 9 months post-partum, T5	9.14%
2 years 9 months post-partum, T6	11.86%
5 years post-partum, T7	12.04%
6 years post-partum, T8	13.21%
8 years post-partum, T9	13.46%
11 years post-partum, T10	12.47%
18 years post-partum, T11	19.83%
Maternal depression frequency	
Never	53.81%
1–4 episodes	37.01%
5–11 episodes	9.13%

a Sample numbers not shown because percentages are based on imputed data (*N* = 8425).

b EPDS item 10: “*The thought of harming myself has occurred to me”* sometimes or often versus never or hardly.

c EPDS score of 13 points or higher, indicating moderate depression.

Consistent with prior analyses (Gordon et al., 2019a; Putnam et al., 2017), we defined depression categorically using a threshold of 13 or higher on the EPDS, which has previously been shown to have high specificity (95.7%) and good predictive validity (66.7%) for major depressive disorder (Murray and Carothers, 1990). Dichotomous variables for each of the 11 time points indicated the presence or absence of maternal depression and the presence or absence of maternal self-harm ideation. Consistent with prior work (Netsi et al., 2018) and to maintain adequate statistical power in each group, ordinal variables indicated the number of timepoints the mother endorsed self-harm ideation and depression: (0) never, (1) between one to four times, and (2) five or more times for self-harm ideation and depression.

To examine the interaction between maternal depression and self-harm ideation, an ordinal variable reflecting each of the four possible combinations of the two variables was created for each of the 11 time points: (0) neither self-harm ideation nor depression, (1) depression but no self-harm ideation, (2) self-harm ideation but no depression, and (3) both depression and self-harm ideation.

We choose the initial comparisons of the self-harm ideation item with how the EPDS is most commonly used, a score of 13 or higher. In contrast to our binary MDD exposure variable, the self-harm ideation variable was based on a single item, thus potentially increasing the likelihood of measurement error. In order to examine this possibility, we therefore conducted a series of sensitivity analyses comparing our main findings with those obtained with models using another single item from the EPDS, item 8 *“I have felt sad or miserable”*. Mothers were categorised into responses of “mostly” or “quite often” versus “hardly ever” or “never” on this item. Analyses for the interaction between maternal self-harm ideation and depression were conducted using this EPDS item instead of the cut-off for major depression.

### Ascertainment of offspring major depressive disorder and self-harm

Offspring major depressive episode and self-harm were assessed at the age 24 using the self-administered computerized version of the Clinical Interview Schedule – Revised (CIS-R)(Lewis et al., 1992). The CIS-R assesses symptoms across multiple domains, and computer algorithms are used to identify past year psychiatric disorders according to ICD-10 diagnostic criteria. The computerised version has demonstrated close agreement with interviewer assessment (Bell et al., 2005; Patton et al., 1999). Two binary outcome variables were derived indicating whether at age 24 the child had (i) ever self-harmed and (ii) met ICD-10 criteria for mild or more severe Major Depressive Disorder (MDD) in the past year.

### Potential confounding variables

Analyses controlled for the highest level of maternal education achieved at 32 weeks’ gestation, maternal age, and maternal marital status (married versus not married), which have all been shown to influence both the exposures (Gavin et al., 2011; Giallo et al., 2018; Howard et al., 2011) and outcomes (Page et al., 2014; Pearson et al., 2013). Maternal education categories were (0) O-level or lower, (1) A-level, (2) university degree.

### Statistical analysis

Binary logistic regression was used to examine associations between maternal self-harm ideation and depression with offspring age 24 depression and self-harm. Logistic regression was also used to assess associations between the timing of maternal self-harm ideation at each of the 11 time points, from 8 weeks’ gestation to 18 years post-partum, with offspring outcome. Mothers with neither depression nor self-harm ideation served as the reference group. All analyses controlled for maternal education, age, and marital status.

These analyses were repeated for each exposure-outcome combination using all available data to examine the potential impact of missingness on results. A priori level of statistical significance was set at 0.05 and all tests were 2-sided. Analyses were conducted using Stata version 16 (Stata Corp, 2019) .

### Missing data

The pattern of missing data in the study sample are presented in eTable 1. A total of 8425 mothers had self-harm ideation data for at least one of the eleven time points. The mean number of timepoints for which these 8425 mothers had self-harm data was 9.54 (*SE* = 0.02). For a total of 2914 mothers self-harm ideation data was available at all eleven timepoints. There was no indication in the pattern of missingness in individual EPDS items that the self-harm ideation question was more often missing than others.

Analyses were conducted on an imputed dataset based on mothers with valid self-harm ideation data for at least one time point (*N* = 8425). Multiple imputation by chained equations (Azur et al., 2011; Royston and White, 2011) was used to generate 50 imputed datasets for each variable. This method assumes that data are missing at random, whereby any systematic differences between the missing and the observed values can be explained by differences in observed data. Imputation models included all variables used in the analysis, as well as additional auxiliary variables (see eTable 1). These were indicators of socioeconomic adversity and correlates of the outcome variables such as offspring depression and self-harm collected earlier in the study. Comparisons of the imputed and non-imputed samples are presented in the online supplement (eTable 1).

## Results

Of the 8425 mothers in the study sample, the majority were married at the time of the child's birth (80.92%) and 48.72% of the offspring were female (Table 1). 16.67% of mothers reported self-harm thoughts on at least one occasion, while nearly half (46.14%) had met criteria for depression at least once. Mothers with self-harm ideation at one or more of the eleven timepoints were generally more socioeconomically disadvantaged, experienced partner abuse, and a range of negative childhood experiences (eTable 2). The prevalence of both maternal self-harm ideation (5.40%) and depression (19.83%) were highest at 18 years post-partum (Table 1). Maternal self-harm ideation without concurrent major depression was rare (range 0.18% to 4.66%) at each timepoint (eTable 10). At each time point, mothers with both self-harm thoughts and major depressive disorder also reported a greater frequency of depressive symptoms. In addition, mothers with self-harm ideation but who did not meet major depressive disorder criteria consistently reported experiencing a greater frequency of depressive symptoms compared to mothers who did not report self-harm ideation nor depression (eTable 3).

**Table 2:** Logistic regressions predicting age 24 offspring self-harm from chronicity of maternal self-harm ideation a and major depressive disorder b (*N* = 8425).

	Offspring self-harm, age 24			Offspring self-harm, age 24	
Predictor variable	AOR (95% CI)	*P* value	Predictor variable	AOR (95% CI)	*P* value
Maternal self-harm ideation frequency a			Maternal depression frequency b		
Never any self-harm ideation	1 (Reference)	NA	Never depressed	1 (Reference)	NA
1–4 times	1.36 (1.08–1.73)	.010	1–4 times	1.30 (1.10–1.55)	.002
5–11 times	1.55 (0.73–3.29)	.249	5–11 times	1.52 (1.17–1.98)	.002

AOR = adjusted odds ratio. Models adjusted for highest level of maternal education achieved at 32 weeks’ gestation, maternal age, and maternal marital status.

a EPDS item 10: “*The thought of harming myself has occurred to me”* sometimes or often versus never or hardly.

b EPDS score of 13 points or higher, indicating moderate depression.

**Table 3:** Logistic regressions predicting offspring depression from chronicity of maternal self-harm ideation and major depressive disorder (*N* = 8425).

	Offspring depression, age 24			Offspring depression, age 24	
Predictor variable	AOR (95% CI)	*P* value	Predictor variable	AOR (95% CI)	*P* value
Maternal self-harm ideation frequency a			Maternal depression frequency b		
Never any self-harm ideation	1 (Reference)	NA	Never depressed	1 (Reference)	NA
1–4 times	1.51 (1.15–1.97)	.003	1–4 times	1.34 (1.09- 1.64)	.006
5–11 times	3.32 (1.63- 6.76)	<0.001	5–11 times	2.22 (1.62- 3.03)	<0.001

AOR = adjusted odds ratio. Models adjusted for highest level of maternal education achieved at 32 weeks’ gestation, maternal age, and maternal marital status.

a EPDS item 10: “*The thought of harming myself has occurred to me”* sometimes or often versus never or hardly.

b EPDS score of 13 points or higher, indicating moderate depression.

Multivariable logistic regressions indicated a dose-response relationship between the frequencies of maternal self-harm ideation and depression with both offspring outcomes (eFigures 22–25). Young adults whose mothers who had reported self-harm ideation on 5–11 occasions were over 1.5 times as likely to have self-harmed by age 24 (Table 2) and over three times more likely to be depressed at age 24 (OR, 3.32; 95% CI, 1.63- 6.76) (Table 3) than their peers whose mothers had never reported self-harm thoughts. Similar-sized odds ratios were obtained for maternal depression frequency and offspring self-harm. However, risk for offspring depression associated with frequent maternal self-harm thoughts (5–11 time points) was more than twice that of the association with offspring self-harm (adjusted OR for offspring depression: 3.32; 95% CI, 1.63- 6.76; OR, 1.55; 95% CI, 0.73–3.29, respectively).

Next, we investigated whether there was an interaction between the timing of maternal self-harm ideation and depression with offspring self-harm (eFigures 1–11) and depression (eFigures 12–22). The majority of the associations were in the expected direction, such that the presence of self-harm ideation and maternal depression at each of the eleven time points was associated with elevated odds for later offspring depression and self-harm. Evidence for whether the presence of *both* maternal self-harm ideation and depression was worse for offspring mental health outcomes was less convincing. Confidence intervals were wide, reflecting the low numbers of mothers with self-harm ideation who were not depressed. When mothers had both self-harm thoughts and met depression criteria, odds ratios were largest at only three timepoints (range: OR, 1.57; 95% CI, 0.87–2.84 to OR, to 1.79; 95% CI, 1.04–3.08) for offspring self-harm (Table 4), and at seven of 11 timepoints (range: OR, 1.71; 95% CI, 0.97–3.01 to OR, 3.47; 95% CI, 1.79–6.73) for offspring depression (Table 5).

**Table 4:** Logistic regressions examining the interaction between maternal self-harm ideation a and major depression b on offspring self-harm (*N* = 8425).

	Offspring self-harm, age 24	
	AOR (95% CI)	*P* value
18 weeks’ gestation, T1		
Maternal depression but no self-harm ideation	1.42 (1.11–1.81)	.006
Maternal self-harm ideation but no depression	1.07 (0.59–1.94)	.822
Maternal self-harm ideation and depression	1.79 (1.04–3.08)	.036
32 weeks’ gestation, T2		
Maternal depression but no self-harm ideation	1.11 (0.87–1.41)	.402
Maternal self-harm ideation but no depression	1.54 (0.66–3.59)	.313
Maternal self-harm ideation and depression	1.54 (0.84–2.79)	.158
8 weeks post-partum, T3		
Maternal depression but no self-harm ideation	1.31 (0.99–1.72)	.056
Maternal self-harm ideation but no depression	1.41 (0.31–6.37)	.652
Maternal self-harm ideation and depression	1.57 (0.87–2.84)	.131
8 months post-partum, T4		
Maternal depression but no self-harm ideation	1.26 (0.94–1.69)	.121
Maternal self-harm ideation but no depression	1.95 (0.89–4.30)	.097
Maternal self-harm ideation and depression	1.12 (0.62–2.06)	.701
1 year 9 months post-partum, T5		
Maternal depression but no self-harm ideation	1.22 (0.95–1.57)	.119
Maternal self-harm ideation but no depression	0.70 (0.19–2.54)	.585
Maternal self-harm ideation and depression	0.84 (0.43–1.64)	.602
2 years 9 months post-partum, T6		
Maternal depression but no self-harm ideation	1.19 (0.95–1.49)	.139
Maternal self-harm ideation but no depression	2.80 (1.06–7.41)	.038
Maternal self-harm ideation and depression	2.45 (1.54–3.90)	< 0.001
5 years post-partum, T7		
Maternal depression but no self-harm ideation	1.32 (1.02–1.72)	.034
Maternal self-harm ideation but no depression	1.90 (0.50–7.20)	.346
Maternal self-harm ideation and depression	1.17 (0.66–2.08)	.586
6 years post-partum, T8		
Maternal depression but no self-harm ideation	1.21 (0.94–1.54)	.133
Maternal self-harm ideation but no depression	0.96 (0.13–6.82)	.966
Maternal self-harm ideation and depression	1.22 (0.63–2.35)	.558
8 years post-partum, T9		
Maternal depression but no self-harm ideation	1.44 (1.12–1.84)	.004
Maternal self-harm ideation but no depression	1.75 (0.53–5.85)	.360
Maternal self-harm ideation and depression	1.47 (0.84–2.57)	.177
11 years post-partum, T10		
Maternal depression but no self-harm ideation	1.34 (1.04–1.72)	.023
Maternal self-harm ideation but no depression	3.07 (0.85–11.12)	.088
Maternal self-harm ideation and depression	2.85 (1.60–5.08)	<0.001
18 years post-partum, T11		
Maternal depression but no self-harm ideation	1.31 (1.03–1.65)	.025
Maternal self-harm ideation but no depression	0.82 (0.27–2.49)	.726
Maternal self-harm ideation and depression	1.07 (0.64–1.80)	.794

AOR = adjusted odds ratio. Models adjusted for highest level of maternal education achieved at 32 weeks’ gestation, maternal age, and maternal marital status. Mothers with neither depression nor self-harm ideation served as the reference group.

a EPDS item 10: “*The thought of harming myself has occurred to me”* sometimes or often versus never or hardly.

b EPDS score of 13 points or higher, indicating moderate depression.

**Table 5:** Logistic regressions examining the interaction between maternal self-harm ideation a and major depressive disorder b on offspring major depressive disorder (*N* = 8425).

	Offspring depression, age 24	
	AOR (95% CI)	*P* value
18 weeks’ gestation, T1		
Maternal depression but no self-harm ideation	1.62 (1.20–2.18)	.002
Maternal self-harm ideation but no depression	0.85 (0.37–1.95)	.700
Maternal self-harm ideation and depression	1.92 (0.98–3.76)	.057
32 weeks’ gestation, T2		
Maternal depression but no self-harm ideation	1.45 (1.08–1.94)	.013
Maternal self-harm ideation but no depression	2.43 (0.96–6.20)	.062
Maternal self-harm ideation and depression	2.54 (1.40–4.61)	.002
8 weeks post-partum, T3		
Maternal depression but no self-harm ideation	1.51 (1.09–2.11)	.015
Maternal self-harm ideation but no depression	2.51 (0.56–11.14)	.225
Maternal self-harm ideation and depression	2.30 (1.06–4.97)	.035
8 months post-partum, T4		
Maternal depression but no self-harm ideation	1.53 (1.04–2.25)	.031
Maternal self-harm ideation but no depression	0.72 (0.16–3.36)	.680
Maternal self-harm ideation and depression	2.02 (1.08–3.79)	.028
1 year 9 months post-partum, T5		
Maternal depression but no self-harm ideation	1.64 (1.18–2.28)	.004
Maternal self-harm ideation but no depression	1.11 (0.24–5.09)	.890
Maternal self-harm ideation and depression	2.15 (1.03–4.47)	.040
2 years 9 months post-partum, T6		
Maternal depression but no self-harm ideation	1.46 (1.07–1.98)	.018
Maternal self-harm ideation but no depression	1.57 (0.37–6.73)	.543
Maternal self-harm ideation and depression	2.11 (1.10–4.05)	.026
5 years post-partum, T7		
Maternal depression but no self-harm ideation	1.46 (1.07–1.99)	.019
Maternal self-harm ideation but no depression	2.17 (0.49–9.54)	.305
Maternal self-harm ideation and depression	1.99 (1.08–3.69)	.029
6 years post-partum, T8		
Maternal depression but no self-harm ideation	1.29 (0.97–1.74)	.084
Maternal self-harm ideation but no depression	1.30 (9.82 × 10^−57^- 1.74 × 10^56^)	.997
Maternal self-harm ideation and depression	1.92 (0.91–4.04)	.086
8 years post-partum, T9		
Maternal depression but no self-harm ideation	1.73 (1.30–2.29)	<0.001
Maternal self-harm ideation but no depression	1.44 (0.22–9.38)	.697
Maternal self-harm ideation and depression	1.43 (0.65–3.14)	.367
11 years post-partum, T10		
Maternal depression but no self-harm ideation	1.51 (1.10–2.07)	.011
Maternal self-harm ideation but no depression	2.34 (0.48–11.30)	.290
Maternal self-harm ideation and depression	3.47 (1.79–6.73)	<0.001
18 years post-partum, T11		
Maternal depression but no self-harm ideation	1.31 (0.98–1.77)	.071
Maternal self-harm ideation but no depression	Empty cell	–
Maternal self-harm ideation and depression	1.71 (0.97–3.01)	.062

AOR = adjusted odds ratio. Models adjusted for highest level of maternal education achieved at 32 weeks gestation, maternal age, and maternal marital status. Mothers with neither depression nor self-harm ideation served as the reference group.

a EPDS item 10: “*The thought of harming myself has occurred to me”* sometimes or often versus never or hardly.

b EPDS score of 13 points or higher, indicating moderate depression.

In terms of timing, the strongest associations between maternal self-harm ideation with (OR, 2.85; 95% CI, 1.60–5.08) and without (OR, 3.07; 95% CI, 0.85–11.12) maternal depression emerged with offspring self-harm at 11 years post-partum (Table 4). Maternal self-harm with (OR, 3.47; 95% CI, 1.79–6.73) and without (OR, 2.34; 95% CI, 0.48–11.30) depression at 11 years post-partum also had the largest association with offspring depression (Table 5).

Results using the unimputed sample of mothers with incomplete self-harm ideation were substantively consistent (eTables 4–7) with those obtained using the imputed sample. Imputed results were more precise as evidenced by the smaller confidence intervals. Results from sensitivity analyses of the interaction between maternal self-harm ideation and depression using the sad/miserable item were largely consistent (eTables 8–9) with those obtained using the cut-off for major depressive disorder.

## Discussion

Over the 17 years that the mothers in this study were followed, nearly 17% reported having thought of harming themselves sometimes or often; the vast majority of these mothers also met criteria for major depression. Self-harm thoughts peaked when the index child was 18 years of age, and we identified the existence of a dose-response relationship between maternal self-harm thoughts and maternal depression with both offspring mental health outcomes. These associations did not appear to be specific, in that maternal self-harm ideation was not more strongly associated with offspring self-harm, and the same was true for depression. In fact, the risk for offspring depression from maternal self-harm thoughts was higher than for maternal depression, suggesting that self-harm ideation may have been accompanied by other changes in the environment that may have triggered subsequent dysphoria in the child. There are a number of possible exposures that might fall into this category, including witnessing traumatising acts of maternal self-harm and associated adversities (e.g., domestic violence, poverty and childhood neglect). Further research should examine whether these variables confound or mediate the association between maternal self-harm ideation and subsequent offspring depression. Furthermore, future studies should use more sophisticated longitudinal modelling techniques to examine how the timing of maternal self-harm ideation and depression influence offspring outcomes, as these data points are correlated.

One potential mechanism for the relationship between maternal self-harm ideation (Gordon et al., 2019b) and offspring depression (Kasamatsu et al., 2019; Raine et al., 2019) in early childhood is through impairments to parenting, particularly mother-infant interactions. Maternal but not paternal dysfunctional personality traits have also been found to be associated with offspring self-harm (Pearson et al., 2018), further suggesting a role of parenting, as mothers typically engage in a larger share of child rearing. Support for the role of parenting in this link is also provided by some evidence that treating maternal depression but not targeting mother-child interactions or parenting is insufficient for improving child outcomes (Letourneau et al., 2017), and may depend on other important factors such as experiences of child abuse which is commoner in the mothers with self-harm, and may be commoner in their children as well.

Another possible explanatory mechanism is via the bi-directional relationship between maternal and offspring psychopathology, which may be partially mediated by direct and indirect genetic effects. Firstly, we observed the greatest peak in maternal self-harm ideation at offspring age 18, an age where depression has commonly already had its onset in adolescents. This onset of adolescence depression could be underlying the peak of maternal self-harm ideation and may support the idea of a bi-directional relationship where mothers have ideas of self-harm because their child is depressed and vice versa. This may have roots in direct and indirect genetic effects.

### Limitations

Mothers, particularly in the perinatal period, may have been reluctant to report their thoughts of self-harm. Second, participant loss at follow-up may have biased all available data analyses. However, substantive findings were similar using the imputed dataset, suggesting that the impact of this bias was minimal. We also assumed that our data were missing at random, and to address this we used auxiliary data in our multiple imputation. There were differences between participants with missing data on several indices of socio-economic disadvantage, which may further limit generalisability of our results. Due to low numbers at each time point, we did not include measures of maternal self-harm behaviour, which has been found to increase risk for offspring depression and self-harm (Geulayov et al., 2014).

Although maternal self-harm ideation conferred risk for both offspring outcomes, from a public health perspective, mothers with depression should still be focused on, as they represent a larger proportion of the population. Self-harm ideation is a component of the EPDS, which is already widely used in clinical practice. Clinicians may consider giving more weight to endorsements of this item as a way of identifying which offspring may be at greatest future risk for mental health problems. Future research should explore mediating pathways and include data on the father as well. Future research could also specifically examine the risk factors for maternal self-harm ideation and explore if self-harm thoughts earlier in life are a risk factor for later maternal self-harm ideation and depression.

## Declaration of Competing Interest

All authors declare no conflicts of interest.

## Appendix Supplementary materials

Image, application 1
