# Supplementary material for: Maternal thoughts of self-harm and their association with future offspring mental health problems
Source: J Affect Disord. 2021 Oct 1;293:422–8. doi: 10.1016/j.jad.2021.06.058 (PMC8370273; doi:10.1016/j.jad.2021.06.058)

**eTable S1. Comparison of those with complete and incomplete maternal self-harm ideation (SHI) ^a^ data**

| **Variable** | | **No.** | **Incomplete SHI data** | **Complete SHI data**  **No. 2,914** | ***P* value** |
| --- | --- | --- | --- | --- | --- |
|  | |  | **Mean (SD) or %** | |  |
| Child sex | Child female | 4,105 | 47.94% | 50.21% | .048 |
| Duration of breastfeeding | Child not breastfed | 1,824 | 26.15% | 15.75% | < .001 |
|  | < 3 months | 1,856 | 23.79% | 21.18% |  |
|  | 3-5 months | 1,402 | 16.85% | 18.05% |  |
|  | 6 months or longer | 3,035 | 33.22% | 45.02% |  |
| Maternal age in years | | 8,425 | 27.74 (4.65) | 29.14 (4.27) | < .001 |
| Mother is married | | 6,774 | 78.22% | 86.44% | < .001 |
| Household overcrowding | > 1 person per room | 341 | 5.42% | 1.81% | < .001 |
| Parity | First born | 3,776 | 44.17% | 47.76% | < .001 |
|  | Second born | 2,980 | 35.50% | 36.49% |  |
|  | Third or higher | 1,558 | 20.33% | 15.75% |  |
| Affected by financial problems during pregnancy | | 821 | 11.85% | 8.34% | < .001 |
| Homeowner | | 6,865 | 77.75% | 89.94% | < .001 |
| Opinion of neighbourhood | Good/very good | 7,843 | 92.46% | 95.19% | < .001 |
|  | Not good/not very good | 554 | 7.54% | 4.81% |  |
| Paternal education | O-levels or lower | 4,121 | 55.10% | 41.91% | < .001 |
|  | A level | 2,326 | 27.69% | 29.97% |  |
|  | University degree | 1,717 | 17.20% | 28.12% |  |
| Maternal education | O-levels or lower | 4,972 | 65.33% | 47.82% | .071 |
|  | A level | 2,130 | 22.60% | 30.62% |  |
|  | University degree | 1,289 | 12.07% | 21.56% |  |
| Mother’s mother had history of depression | | 1,646 | 20.30% | 18.65% |  |
| Pregnancy was intentional | | 6,180 | 70.94% | 78.82% | <.001 |
| Partner emotional abuse during pregnancy | | 380 | 5.48% | 3.91% | .002 |
| Mother was sexually abused as child (age < 17) | | 368 | 4.88% | 3.48% | .003 |
| Mother was emotionally abused by a parent as a child (age < 17) | | 613 | 7.77% | 6.48% | .030 |
| Mother was physically abused by a parent as a child (age < 17) | | 249 | 3.37% | 2.24% | .004 |
| Offspring major depression, age 24 | | 314 | 11.46 | 9.66% | .110 |
| Offspring self-harm, age 24 | | 625 | 20.85% | 20.86% | .998 |

^a^ EPDS item 10: “*The thought of harming myself has occurred to me”* sometimes or often versus never or hardly.

^b^ EPDS score of 13 points or higher, indicating moderate depression.

**eTable 1 continued. Comparison of those with complete and incomplete maternal self-harm ideation (SHI) ^a^ data**

| **Variable** | **No.** | **Incomplete SHI data** | **Complete SHI data**  **No. 2,914** | ***P* value** |
| --- | --- | --- | --- | --- |
|  |  | **Mean (SD) or %** | |  |
| Maternal self-harm ideation timing ^a^ |  |  |  |  |
| 18 weeks gestation, T1 | 7,884 | 4.91% | 2.81% | <.001 |
| 32 weeks gestation, T2 | 8,220 | 2.36% | 1.92% | .200 |
| 8 weeks post-partum, T3 | 8,397 | 1.88% | 0.86% | <.001 |
| 8 months post-partum, T4 | 8,400 | 2.35% | 2.02% | .335 |
| 1 year 9 months post-partum, T5 | 8,389 | 2.45% | 1.65% | .017 |
| 2 years 9 months post-partum, T6 | 8,374 | 2.99% | 2.09% | .016 |
| 5 years post-partum, T7 | 7,392 | 2.95% | 1.65% | <.001 |
| 6 years post-partum, T8 | 7,131 | 2.42% | 1.37% | .002 |
| 8 years post-partum, T9 | 6,398 | 2.61% | 1.92% | .067 |
| 11 years post-partum, T10 | 6,050 | 1.75% | 1.54% | .523 |
| 18 years post-partum, T11 | 3,719 | 6.09% | 4.19% | .023 |
| Maternal self-harm ideation frequency ^a^ |  |  |  |  |
| Never any self-harm ideation | 7,242 | 85.59% | 86.65% | .335 |
| 1-4 times | 1,134 | 13.77% | 12.87% |  |
| 5-11 times | 49 | 0.64% | 0.48% |  |
| Maternal depression timing ^b^ |  |  |  |  |
| 18 weeks gestation, T1 | 7,820 | 11.98% | 8.66% | <.001 |
| 32 weeks gestation, T2 | 8,233 | 14.46% | 10.30% | <.001 |
| 8 weeks post-partum, T3 | 8,418 | 9.52% | 7.04% | <.001 |
| 8 months post-partum, T4 | 8,420 | 8.66% | 6.79% | .003 |
| 1 year 9 months post-partum, T5 | 8,416 | 9.91% | 7.69% | .001 |
| 2 years 9 months post-partum, T6 | 8,379 | 12.92% | 9.85% | <.001 |
| 5 years post-partum, T7 | 7,351 | 13.06% | 10.06% | <.001 |
| 6 years post-partum, T8 | 7,090 | 14.44% | 10.91% | <.001 |
| 8 years post-partum, T9 | 6,341 | 13.43% | 10.62% | .001 |
| 11 years post-partum, T10 | 6,008 | 13.34% | 10.36% | <.001 |
| 18 years post-partum, T11 | 3,691 | 21.90% | 16.74% | .001 |
| Maternal depression frequency ^b^ |  |  |  |  |
| Never depressed | 4,922 | 58.32% | 58.61% | .900 |
| 1-4 times | 2,945 | 35.11% | 34.66% |  |
| 5-11 times | 558 | 6.57% | 6.73% |  |

^a^ EPDS item 10: “*The thought of harming myself has occurred to me”* sometimes or often versus never or hardly.

^b^ EPDS score of 13 points or higher, indicating moderate depression.

**eTable 2. Comparison of mothers with at least one episode of self-harm ideation (SHI) ^a^ to those with no episodes (*N* = 8,425)**

| **Variable** | | **Never any SHI** | **One or more SHI** | ***P* value** |
| --- | --- | --- | --- | --- |
|  |  | **Mean (SD) or %** | |  |
| Child is female | | 48.62% | 49.22% | .721 |
| Duration of breastfeeding | Child not breastfed | 21.73% | 27.07% | <.001 |
|  | < 3 months | 22.90% | 23.12% |  |
|  | 3-5 months | 17.04% | 18.41% |  |
|  | 6 months or longer | 38.33% | 31.38% |  |
| Maternal age in years | | 28.40 (0.06) | 27.35 (0.14) | <.001 |
| Mother is married | | 82.34% | 73.85% | <.001 |
| Household overcrowding | > 1 person per room | 3.66% | 7.12% | <.001 |
| Parity | First born | 44.90% | 47.85% | .015 |
|  | Second born | 36.60% | 32.04% |  |
|  | Third or higher | 18.49% | 20.11% |  |
| Affected by financial problems during pregnancy | | 9.57% | 16.67% | <.001 |
| Homeowner | | 83.71% | 72.92% | <.001 |
| Opinion of neighbourhood | Good/very good | 94.01% | 90.40% | <.001 |
|  | Not good/not very good | 5.99% | 9.60% |  |
| Paternal education | O-levels or lower | 49.72% | 58.04% | <.001 |
|  | A level | 29.00% | 24.50% |  |
|  | University degree | 21.27% | 17.47% |  |
| Maternal education | O-levels or lower | 58.27% | 64.48% | <.001 |
|  | A level | 25.92% | 22.55% |  |
|  | University degree | 15.81% | 12.97% |  |
| Mother’s mother had history of depression | | 17.66% | 26.15% | <.001 |
| Pregnancy was intentional | | 74.88% | 67.64% | <.001 |
| Partner emotional abuse during pregnancy | | 3.95% | 10.76% | <.001 |
| Mother was sexually abused as child (age < 17) | | 3.56% | 8.59% | <.001 |
| Mother was emotionally abused by a parent as a child (age < 17) | | 6.07% | 13.64% | <.001 |
| Mother was physically abused by a parent as a child (age < 17) | | 2.26% | 6.56% | <.001 |
| Offspring major depressive disorder, age 24 | | 10.07% | 16.31% | <.001 |
| Offspring self-harm, age 24 | | 18.35% | 23.93% | .006 |

Sample numbers not shown because percentages are based on imputed data (*N*=8,425).

^a^ EPDS item 10: “*The thought of harming myself has occurred to me”* sometimes or often versus never or hardly.

**eTable 3. Maternal depressive symptoms by self-harm ideation (SHI) ^a^ and major depressive disorder (MDD) ^b^ status in the imputed sample (*N* = 8,425)**

|  | **Neither MDD nor SHI** | **MDD but no SHI** | **SHI but no MDD** | **Both MDD and SHI** |
| --- | --- | --- | --- | --- |
|  | **Mean (SE) maternal depressive symptoms** | | | |
| 18 weeks’ gestation, T1 | 5.35 (0.04) | 14.91 (0.08) | 7.89 (0.20) | 17.42 (0.29) |
| 32 weeks’ gestation, T2 | 5.30 (0.04) | 15.25 (0.08) | 8.59 (0.34) | 18.17 (0.34) |
| 8 weeks post-partum, T3 | 4.86 (0.04) | 15.20 (0.10) | 10.10 (0.37) | 19.23 (0.39) |
| 8 months post-partum, T4 | 4.26 (0.04) | 15.29 (0.10) | 9.93 (0.37) | 18.53 (0.32) |
| 1 year 9 months post-partum, T5 | 4.50 (0.04) | 15.36 (0.10) | 9.35 (0.46) | 18.22 (0.30) |
| 2 years 9 months post-partum, T6 | 4.82 (0.04) | 15.18 (0.08) | 10.38 (0.36) | 19.08 (0.27) |
| 5 years post-partum, T7 | 4.61 (0.04) | 15.17 (0.08) | 9.89 (0.53) | 18.87 (0.31) |
| 6 years post-partum, T8 | 4.79 (0.05) | 15.11 (0.08) | 10.57 (0.46) | 19.54 (0.35) |
| 8 years post-partum, T9 | 4.65 (0.05) | 15.19 (0.09) | 10.11 (0.52) | 20.45 (0.38) |
| 11 years post-partum, T10 | 4.36 (0.05) | 15.38 (0.09) | 9.68 (0.58) | 20.21 (0.46) |
| 18 years post-partum, T11 | 5.61 (0.07) | 15.02 (0.08) | 9.49 (0.45) | 20.06 (0.33) |

^a^ EPDS item 10: “*The thought of harming myself has occurred to me”* sometimes or often versus never or hardly.

^b^ EPDS score of 13 points or higher, indicating moderate depression.

**eTable 4. Logistic regressions predicting offspring self-harm from chronicity of maternal self-harm ideation ^a^ and major depressive disorder ^b^ in the unimputed sample (all available data)**

|  | **Offspring self-harm, age 24** | | |  | **Offspring self-harm, age 24** | | |
| --- | --- | --- | --- | --- | --- | --- | --- |
| **Predictor variable** | **No.** | **AOR (95% CI)** | ***P* value** | **Predictor variable** | **No.** | **AOR (95% CI)** | ***P* value** |
| Maternal self-harm ideation frequency ^a^ | 1,630 |  |  | Maternal depression frequency ^b^ | 1,579 |  |  |
| Never any self-harm ideation |  | 1 (Reference) | NA | Never depressed |  | 1 (Reference) | NA |
| 1-4 times |  | 1.43 (1.00-2.04) | .047 | 1-4 times |  | 1.18 (0.91-1.53) | .205 |
| 5-11 times |  | 2.10 (0.38-11.61) | .397 | 5-11 times |  | 1.42 (0.86-2.34) | .170 |

^a^ EPDS item 10: “*The thought of harming myself has occurred to me”* sometimes or often versus never or hardly.

^b^ EPDS score of 13 points or higher, indicating moderate depression.

AOR = adjusted odds ratio. Models adjusted for highest level of maternal education achieved at 32 weeks gestation, maternal age, and maternal marital status.

**eTable 5. Logistic regressions predicting offspring major depressive disorder from chronicity of** **maternal self-harm ideation ^a^ and major depressive disorder ^b^ in the unimputed sample (all available data)**

|  | **Offspring depression, age 24** | | |  | **Offspring depression, age 24** | | |
| --- | --- | --- | --- | --- | --- | --- | --- |
| **Predictor variable** | **No.** | **AOR (95% CI)** | ***P* value** | **Predictor variable** | **No.** | **AOR (95% CI)** | ***P* value** |
| Maternal self-harm ideation frequency ^a^ | 1,630 |  |  | Maternal depression frequency ^b^ | 1,579 |  |  |
| Never any self-harm ideation |  | 1 (Reference) | NA | Never depressed |  | 1 (Reference) | NA |
| 1-4 times |  | 1.46 (0.92-2.33) | .110 | 1-4 times |  | 1.15 (0.80-1.66) | .462 |
| 5-11 times |  | 18.53 (3.27-105.01) | .001 | 5-11 times |  | 3.18 (1.84-5.52) | <.001 |

^a^ EPDS item 10: “*The thought of harming myself has occurred to me”* sometimes or often versus never or hardly.

^b^ EPDS score of 13 points or higher, indicating moderate depression.

AOR = adjusted odds ratio. Models adjusted for highest level of maternal education achieved at 32 weeks gestation, maternal age, and maternal marital status.

**eTable 6. Logistic regressions predicting offspring self-harm from the timing of maternal self-harm ideation ^a^ and major depressive disorder ^b^ in the unimputed sample (all available data)**

|  | **Offspring self-harm, age 24** | |
| --- | --- | --- |
|  | **AOR (95% CI)** | ***P* value** |
| 18 weeks gestation, T1 |  |  |
| Maternal depression but no self-harm ideation | 1.53 (1.08-2.17 | .018 |
| Maternal self-harm ideation but no depression | 0.97 (0.42-2.26) | .947 |
| Maternal self-harm ideation and depression | 2.09 (1.01-4.32) | .048 |
| 32 weeks gestation, T2 |  |  |
| Maternal depression but no self-harm ideation | 1.18 (0.85-1.65) | .326 |
| Maternal self-harm ideation but no depression | 0.45 (0.06-3.58) | .450 |
| Maternal self-harm ideation and depression | 0.65 (0.27-1.58) | .343 |
| 8 weeks post-partum, T3 |  |  |
| Maternal depression but no self-harm ideation | 1.14 (0.76-1.70) | .538 |
| Maternal self-harm ideation but no depression | Empty cell | --- |
| Maternal self-harm ideation and depression | 2.30 (0.99-5.37) | .054 |
| 8 months post-partum, T4 |  |  |
| Maternal depression but no self-harm ideation | 1.31 (0.84-2.03) | .228 |
| Maternal self-harm ideation but no depression | 1.61 (0.41-6.30) | .497 |
| Maternal self-harm ideation and depression | 1.28 (0.56-2.90) | .558 |
| 1 year 9 months post-partum, T5 |  |  |
| Maternal depression but no self-harm ideation | 1.07 (0.69-1.67) | .762 |
| Maternal self-harm ideation but no depression | 2.06 (0.48-8.84) | .328 |
| Maternal self-harm ideation and depression | 0.63 (0.22-1.86) | .406 |
| 2 years 9 months post-partum, T6 |  |  |
| Maternal depression but no self-harm ideation | 1.30 (0.88-1.92) | .187 |
| Maternal self-harm ideation but no depression | 4.19 (0.83-21.11) | .083 |
| Maternal self-harm ideation and depression | 2.87 (1.40-5.90) | .004 |
| 5 years post-partum, T7 |  |  |
| Maternal depression but no self-harm ideation | 1.44 (1.00-2.08) | .050 |
| Maternal self-harm ideation but no depression | 13.00 (1.34-126.23) | .027 |
| Maternal self-harm ideation and depression | 1.83 (0.78-4.27) | .162 |
| 6 years post-partum, T8 |  |  |
| Maternal depression but no self-harm ideation | 0.97 (0.67-1.40) | .855 |
| Maternal self-harm ideation but no depression | Empty cell | -- |
| Maternal self-harm ideation and depression | 1.96 (0.82-4.69) | .132 |
| 8 years post-partum, T9 |  |  |
| Maternal depression but no self-harm ideation | 1.36 (0.94-1.98) | .103 |
| Maternal self-harm ideation but no depression | 1.31 (0.13-12.74) | .818 |
| Maternal self-harm ideation and depression | 1.86 (0.83-4.17) | .133 |
| 11 years post-partum, T10 |  |  |
| Maternal depression but no self-harm ideation | 1.49 (1.06-2.11) | .023 |
| Maternal self-harm ideation but no depression | 7.47 (0.64-87.09) | .109 |
| Maternal self-harm ideation and depression | 1.42 (0.55-3.65) | .469 |
| 18 years post-partum, T11 |  |  |
| Maternal depression but no self-harm ideation | 1.27 (0.91-1.77) | .163 |
| Maternal self-harm ideation but no depression | 2.23 (0.55-9.04) | .263 |
| Maternal self-harm ideation and depression | 1.95 (1.08-3.50) | .026 |

^a^ EPDS item 10: “*The thought of harming myself has occurred to me”* sometimes or often versus never or hardly.

^b^ EPDS score of 13 points or higher, indicating moderate depression.

AOR = adjusted odds ratio. Models adjusted for highest level of maternal education achieved at 32 weeks gestation, maternal age, and maternal marital status.

**eTable 7. Logistic regressions predicting offspring depression from the timing of maternal self-harm ideation ^a^ and major depressive disorder ^b^ in the unimputed sample (all available data)**

|  | **Offspring depression, age 24** | |
| --- | --- | --- |
|  | **AOR (95% CI)** | ***P* value** |
| 18 weeks gestation, T1 |  |  |
| Maternal depression but no self-harm ideation | 1.63 (1.05-2.51) | .028 |
| Maternal self-harm ideation but no depression | 1.71 (0.69-4.25) | .249 |
| Maternal self-harm ideation and depression | 1.76 (0.74-4.21) | .203 |
| 32 weeks gestation, T2 |  |  |
| Maternal depression but no self-harm ideation | 1.38 (0.91-2.08) | .129 |
| Maternal self-harm ideation but no depression | 2.05 (0.43-9.90) | .370 |
| Maternal self-harm ideation and depression | 1.28 (0.52-3.15) | .590 |
| 8 weeks post-partum, T3 |  |  |
| Maternal depression but no self-harm ideation | 0.99 (0.57-1.73) | .976 |
| Maternal self-harm ideation but no depression | 2.87 (0.24-33.83) | .402 |
| Maternal self-harm ideation and depression | 1.80 (0.65-5.03) | .260 |
| 8 months post-partum, T4 |  |  |
| Maternal depression but no self-harm ideation | 1.26 (0.70-2.25) | .444 |
| Maternal self-harm ideation but no depression | Empty cell | -- |
| Maternal self-harm ideation and depression | 2.81 (1.22-6.46) | .015 |
| 1 year 9 months post-partum, T5 |  |  |
| Maternal depression but no self-harm ideation | 1.48 (0.86-2.54) | .158 |
| Maternal self-harm ideation but no depression | 2.81 (0.55-14.42) | .216 |
| Maternal self-harm ideation and depression | 1.65 (0.56-4.87) | .364 |
| 2 years 9 months post-partum, T6 |  |  |
| Maternal depression but no self-harm ideation | 1.33 (0.80-2.19) | .267 |
| Maternal self-harm ideation but no depression | 1.26 (0.13-11.84) | .842 |
| Maternal self-harm ideation and depression | 2.12 (0.88-5.10) | .093 |
| 5 years post-partum, T7 |  |  |
| Maternal depression but no self-harm ideation | 1.29 (0.79-2.09) | .305 |
| Maternal self-harm ideation but no depression | 2.45 (0.23-25.66) | .456 |
| Maternal self-harm ideation and depression | 2.28 (0.84-6.19) | .105 |
| 6 years post-partum, T8 |  |  |
| Maternal depression but no self-harm ideation | 1.03 (0.63-1.68) | .914 |
| Maternal self-harm ideation but no depression | Empty cell | -- |
| Maternal self-harm ideation and depression | 3.62 (1.44-9.11) | .006 |
| 8 years post-partum, T9 |  |  |
| Maternal depression but no self-harm ideation | 1.95 (1.25-3.04) | .003 |
| Maternal self-harm ideation but no depression | 8.84 (1.17-66.75) | .035 |
| Maternal self-harm ideation and depression | 1.58 (0.54-4.68) | .406 |
| 11 years post-partum, T10 |  |  |
| Maternal depression but no self-harm ideation | 1.75 (1.14-2.70) |  |
| Maternal self-harm ideation but no depression | Empty cell | -- |
| Maternal self-harm ideation and depression | 4.46 (1.79-11.11) | .001 |
| 18 years post-partum, T11 |  |  |
| Maternal depression but no self-harm ideation | 1.15 (0.73-1.83) | .543 |
| Maternal self-harm ideation but no depression | 1.14 (0.14-9.45) | .902 |
| Maternal self-harm ideation and depression | 2.77 (1.41-5.44) | .003 |

^a^ EPDS item 10: “*The thought of harming myself has occurred to me”* sometimes or often versus never or hardly.

^b^ EPDS score of 13 points or higher, indicating moderate depression.

AOR = adjusted odds ratio. Models adjusted for highest level of maternal education achieved at 32 weeks gestation, maternal age, and maternal marital status.

**eTable 8. Logistic regressions examining the interaction between maternal self-harm ideation ^a^ and low mood ^b^ on offspring self-harm (*N* = 8,425)**

|  | **Offspring self-harm, age 24** | |
| --- | --- | --- |
|  | **AOR (95% CI)** | ***P* value** |
| 18 weeks gestation, T1 |  |  |
| Maternal low mood but no self-harm ideation | 1.20 (0.87-1.65) | .264 |
| Maternal self-harm ideation but not low mood | 1.16 (0.54-2.50) | .703 |
| Maternal self-harm ideation and low mood | 1.82 (0.84-3.94) | .132 |
| 32 weeks gestation, T2 |  |  |
| Maternal low mood but no self-harm ideation | 1.26 (0.93-1.70) | .138 |
| Maternal self-harm ideation but not low mood | 0.56 (0.13-2.51) | .451 |
| Maternal self-harm ideation and low mood | 0.65 (0.25-1.70) | .377 |
| 8 weeks post-partum, T3 |  |  |
| Maternal low mood but no self-harm ideation | 1.17 (0.82-1.65) | .381 |
| Maternal self-harm ideation but not low mood | Empty cell | -- |
| Maternal self-harm ideation and low mood | 2.14 (0.93-4.95) | .075 |
| 8 months post-partum, T4 |  |  |
| Maternal low mood but no self-harm ideation | 1.27 (0.88-1.81) | .198 |
| Maternal self-harm ideation but not low mood | 1.84 (0.46-7.47) | .391 |
| Maternal self-harm ideation and low mood | 1.24 (0.55-2.81) | .605 |
| 1 year 9 months post-partum, T5 |  |  |
| Maternal low mood but no self-harm ideation | 1.09 (0.76-1.55) | .638 |
| Maternal self-harm ideation but not low mood | 1.79 (0.44-7.30) | .419 |
| Maternal self-harm ideation and low mood | 0.66 (0.22-1.94) | .450 |
| 2 years 9 months post-partum, T6 |  |  |
| Maternal low mood but no self-harm ideation | 1.34 (0.97-1.84) | .077 |
| Maternal self-harm ideation but not low mood | 4.26 (0.84-21.58) | .080 |
| Maternal self-harm ideation and sad/miserable | 2.92 (1.42-5.99) | .004 |
| 5 years post-partum, T7 |  |  |
| Maternal low mood but no self-harm ideation | 1.39 (1.03-1.88) | .031 |
| Maternal self-harm ideation but not sad/miserable | 4.37 (0.27-70.19) | .298 |
| Maternal self-harm ideation and sad/miserable | 2.33 (1.06-5.13) | .035 |
| 6 years post-partum, T8 |  |  |
| Maternal low mood but no self-harm ideation | 1.10 (0.85-1.42) | .461 |
| Maternal self-harm ideation but not sad/miserable | Empty cell | -- |
| Maternal self-harm ideation and sad/miserable | 1.79 (0.76-4.22) | .182 |
| 8 years post-partum, T9 |  |  |
| Maternal low mood but no self-harm ideation | 1.33 (0.97-1.83) | .078 |
| Maternal self-harm ideation but not sad/miserable | 1.48 (0.15-14.35) | .734 |
| Maternal self-harm ideation and sad/miserable | 1.87 (0.83-4.20) | .129 |
| 11 years post-partum, T10 |  |  |
| Maternal low mood but no self-harm ideation | 1.25 (0.97-1.62) | .090 |
| Maternal self-harm ideation but not sad/miserable | Empty cell | -- |
| Maternal self-harm ideation and sad/miserable | 1.78 (0.76-4.17) | .184 |
| 18 years post-partum, T11 |  |  |
| Maternal low mood but no self-harm ideation | 1.27 (1.00-1.62) | .053 |
| Maternal self-harm ideation but not sad/miserable | Empty cell | -- |
| Maternal self-harm ideation and sad/miserable | 1.91 (1.09-3.38) | .025 |

^a^ EPDS item 10: “*The thought of harming myself has occurred to me”* sometimes or often versus never or hardly.

^b^ EPDS item 8: “*I have felt sad or miserable in the past week*” sometimes or often versus never or hardly.

AOR = adjusted odds ratio. Models adjusted for highest level of maternal education achieved at 32 weeks gestation, maternal age, and maternal marital status. Mothers with neither low mood nor self-harm ideation served as the reference group.

**eTable 9. Logistic regressions examining the interaction between maternal self-harm ideation ^a^ and low mood ^b^ on offspring major depressive disorder (*N* = 8,425)**

|  | **Offspring depression, age 24** | |
| --- | --- | --- |
|  | **AOR (95% CI)** | ***P* value** |
| 18 weeks gestation, T1 |  |  |
| Maternal low mood but no self-harm ideation | 1.17 (0.77-1.76) | .458 |
| Maternal self-harm ideation but not sad/miserable | 1.70 (0.72-4.01) | .224 |
| Maternal self-harm ideation and sad/miserable | 1.63 (0.64-4.13) | .302 |
| 32 weeks gestation, T2 |  |  |
| Maternal low mood but no self-harm ideation | 1.30 (0.88-1.91) | .189 |
| Maternal self-harm ideation but not sad/miserable | 1.79 (0.50-6.46) | .372 |
| Maternal self-harm ideation and sad/miserable | 1.25 (0.47-3.34) | .654 |
| 8 weeks post-partum, T3 |  |  |
| Maternal low mood but no self-harm ideation | 0.91 (0.56-1.48) | .705 |
| Maternal self-harm ideation but not sad/miserable | 7.02 (0.40-124.22) | .184 |
| Maternal self-harm ideation and sad/miserable | 1.66 (0.60-4.59) | .333 |
| 8 months post-partum, T4 |  |  |
| Maternal low mood but no self-harm ideation | 1.09 (0.66-1.78) | .741 |
| Maternal self-harm ideation but not sad/miserable | 1.03 (0.12-8.49) | .978 |
| Maternal self-harm ideation and sad/miserable | 2.24 (0.94-5.33) | .067 |
| 1 year 9 months post-partum, T5 |  |  |
| Maternal low mood but no self-harm ideation | 1.71 (1.12-2.62) | .014 |
| Maternal self-harm ideation but not sad/miserable | 1.08 (0.13-8.88) | .941 |
| Maternal self-harm ideation and sad/miserable | 2.37 (0.87-6.44) | .090 |
| 2 years 9 months post-partum, T6 |  |  |
| Maternal low mood but no self-harm ideation | 0.92 (0.58-1.46) | .718 |
| Maternal self-harm ideation but not sad/miserable | 1.19 (0.13-10.88) | .880 |
| Maternal self-harm ideation and sad/miserable | 2.02 (0.84-4.89) | .117 |
| 5 years post-partum, T7 |  |  |
| Maternal low mood but no self-harm ideation | 1.21 (0.81-1.81) | .348 |
| Maternal self-harm ideation but not sad/miserable | 12.47 (0.77-201.94) | .182 |
| Maternal self-harm ideation and sad/miserable | 1.97 (0.73-5.32) | .182 |
| 6 years post-partum, T8 |  |  |
| Maternal low mood but no self-harm ideation | 1.06 (0.75-1.51) | .734 |
| Maternal self-harm ideation but not sad/miserable | Empty cell | -- |
| Maternal self-harm ideation and sad/miserable | 3.39 (1.36-8.40) | .009 |
| 8 years post-partum, T9 |  |  |
| Maternal low mood but no self-harm ideation | 1.30 (0.85-1.99) | .218 |
| Maternal self-harm ideation but not sad/miserable | 11.14 (1.54-80.58) | .017 |
| Maternal self-harm ideation and sad/miserable | 1.46 (0.49-4.35) | .491 |
| 11 years post-partum, T10 |  |  |
| Maternal low mood but no self-harm ideation | 1.32 (0.94-1.86) | .113 |
| Maternal self-harm ideation but not sad/miserable | Empty cell | -- |
| Maternal self-harm ideation and sad/miserable | 3.75 (1.54-9.16) | .004 |
| 18 years post-partum, T11 |  |  |
| Maternal low mood but no self-harm ideation | 1.02 (0.73-1.43) | .906 |
| Maternal self-harm ideation but not sad/miserable | 6.25 (0.36-108.80) | .209 |
| Maternal self-harm ideation and sad/miserable | 2.37 (1.21-4.64) | .011 |

^a^ EPDS item 10: “*The thought of harming myself has occurred to me”* sometimes or often versus never or hardly.

^b^ EPDS item 8: “*I have felt sad or miserable in the past week*” sometimes or often versus never or hardly.

AOR = adjusted odds ratio. Models adjusted for highest level of maternal education achieved at 32 weeks gestation, maternal age, and maternal marital status. Mothers with neither low mood nor self-harm ideation served as the reference group.

**eTable 10. Distribution ^a^ of maternal self-harm ideation (SHI) ^b^ and major depressive disorder (MDD) ^c^ at each time point (*N* = 8,425)**

|  | **Neither MDD nor SHI** | **MDD but no SHI** | **SHI but no MDD** | **Both MDD and SHI** |
| --- | --- | --- | --- | --- |
| 18 weeks’ gestation, T1 | 86.74% | 9.03% | 2.20% | 2.03% |
| 32 weeks’ gestation, T2 | 86.16% | 11.60% | 0.70% | 1.54% |
| 8 weeks post-partum, T3 | 91.08% | 7.40% | 2.61% | 1.26% |
| 8 months post-partum, T4 | 91.52% | 6.25% | 4.66% | 1.77% |
| 1 year 9 months post-partum, T5 | 90.48% | 7.36% | 0.38% | 1.79% |
| 2 years 9 months post-partum, T6 | 87.77% | 9.53% | 0.37% | 2.33% |
| 5 years post-partum, T7 | 87.71% | 9.77% | 0.25% | 2.27% |
| 6 years post-partum, T8 | 86.60% | 11.24% | 0.18% | 1.98% |
| 8 years post-partum, T9 | 87.31% | 10.20% | 0.23% | 2.26% |
| 11 years post-partum, T10 | 87.32% | 10.77% | 2.06% | 1.70% |
| 18 years post-partum, T11 | 79.68% | 14.93% | 0.49% | 4.91% |

^a^ Sample numbers not shown because percentages are based on imputed data (*N*=8,425).

^b^ EPDS item 10: “*The thought of harming myself has occurred to me”* sometimes or often versus never or hardly.

^c^ EPDS score of 13 points or higher, indicating moderate depression.

**eFigures 1-11. Proportion of offspring with age 24 self-harm by frequency of maternal self-harm ideation (SHI) and major depressive disorder (MDD) (N = 8,425).**

**
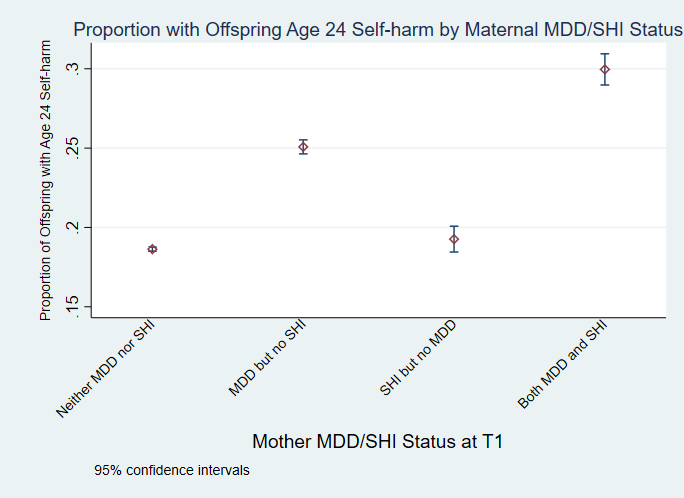

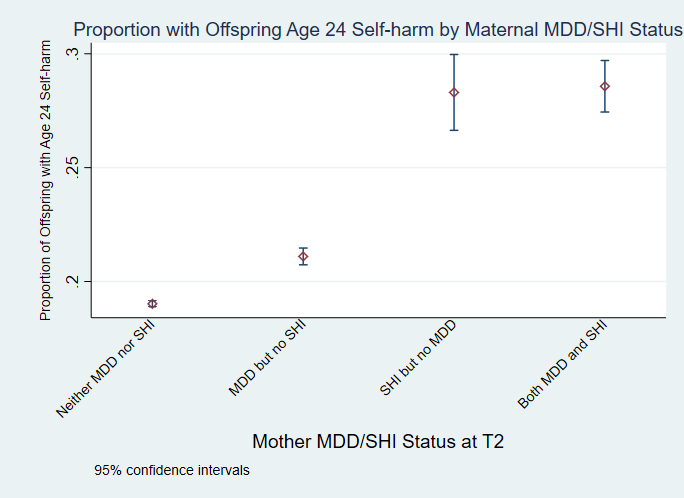

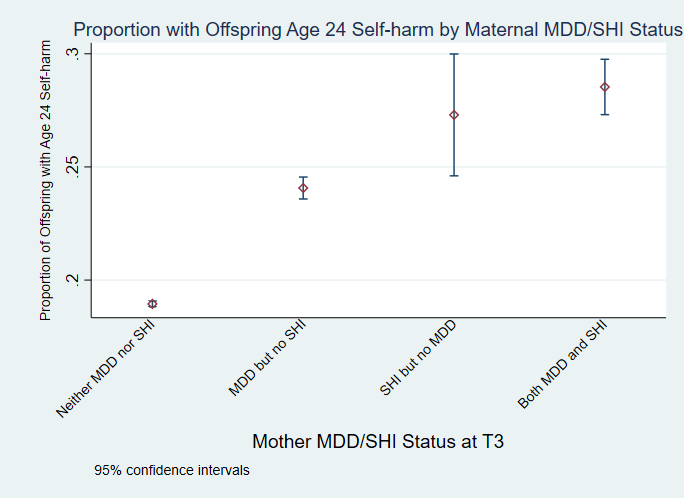

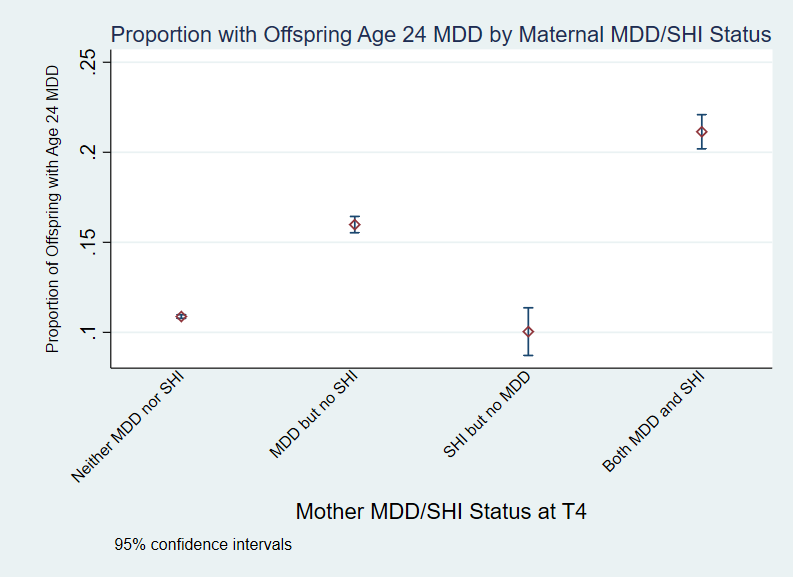
**

**
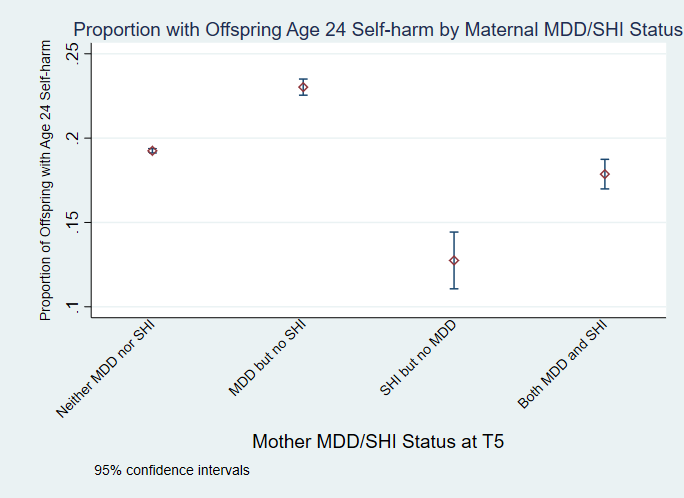

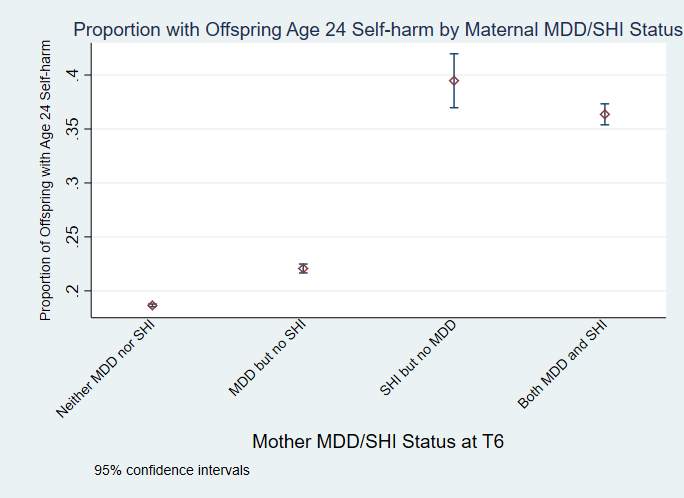

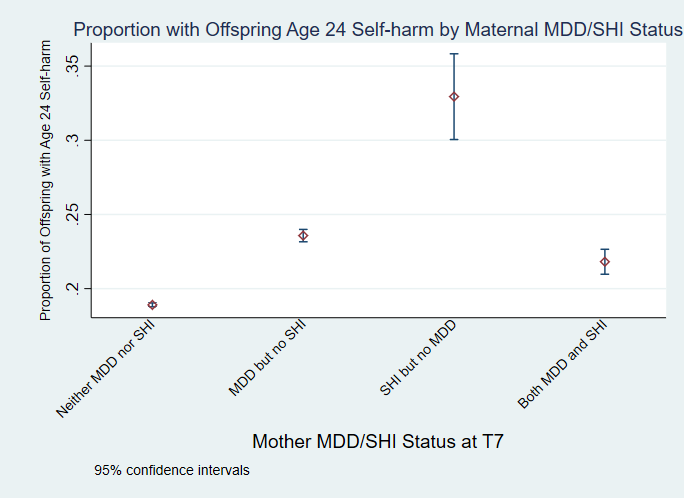

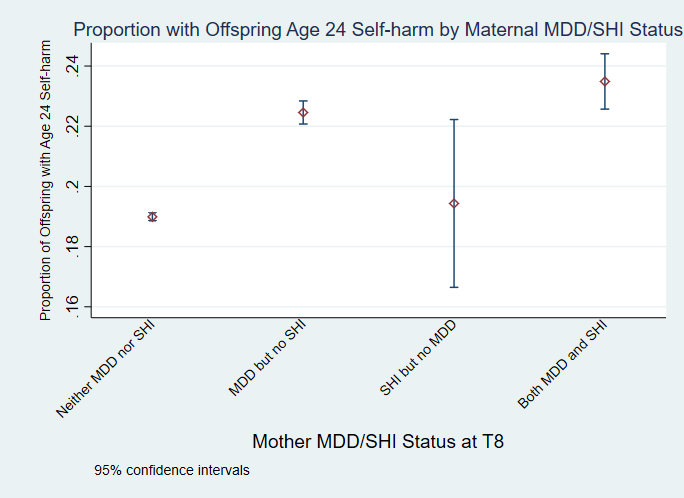

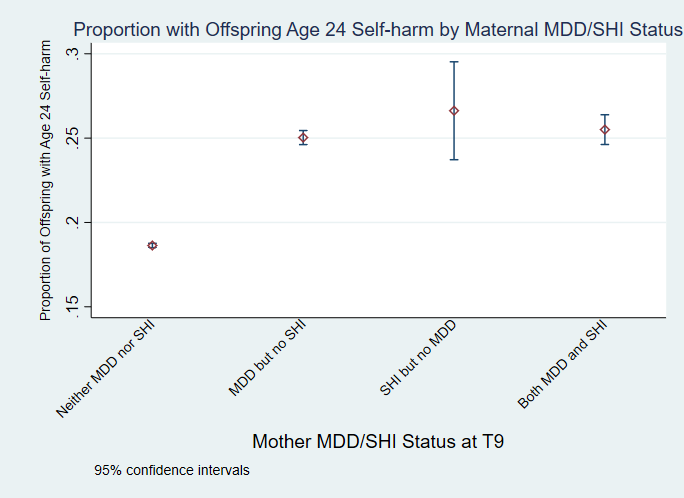

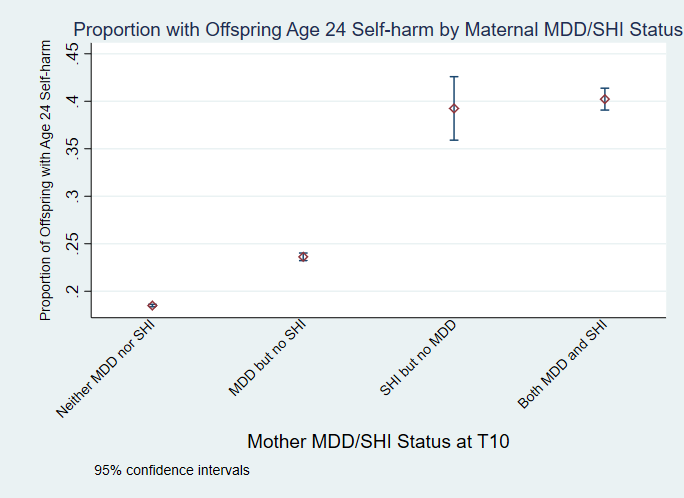

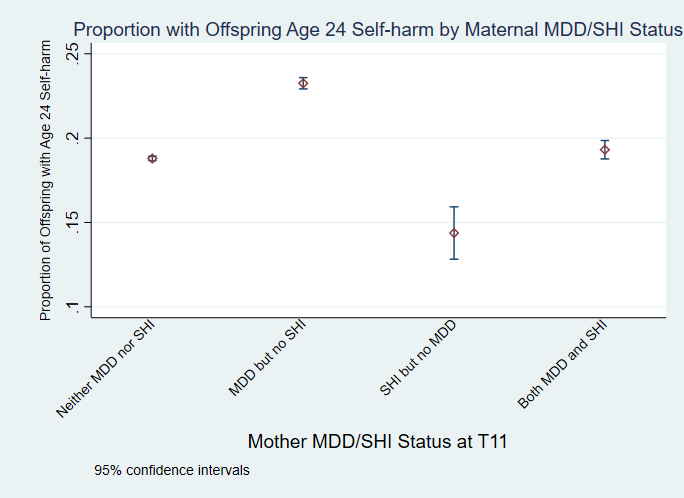
**

**eFigures 12-22. Proportion of offspring with age 24 depression by frequency of maternal self-harm ideation (SHI) and major depressive disorder (MDD) (N = 8,425).**

**
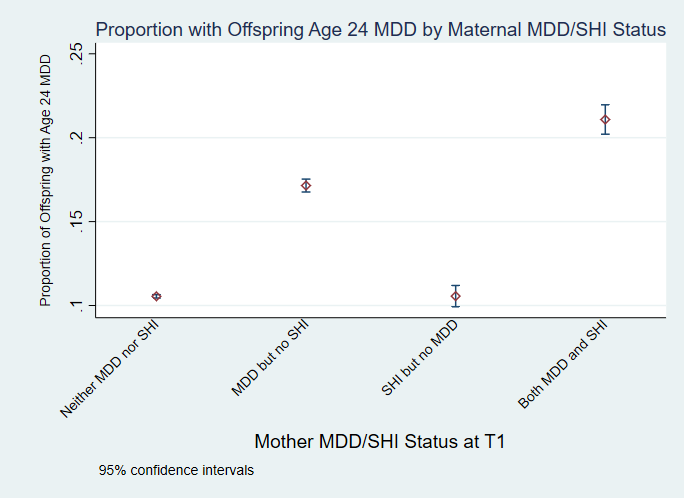

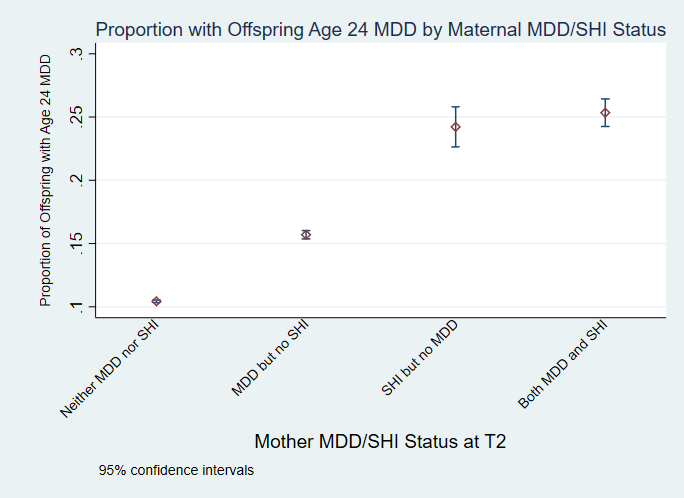

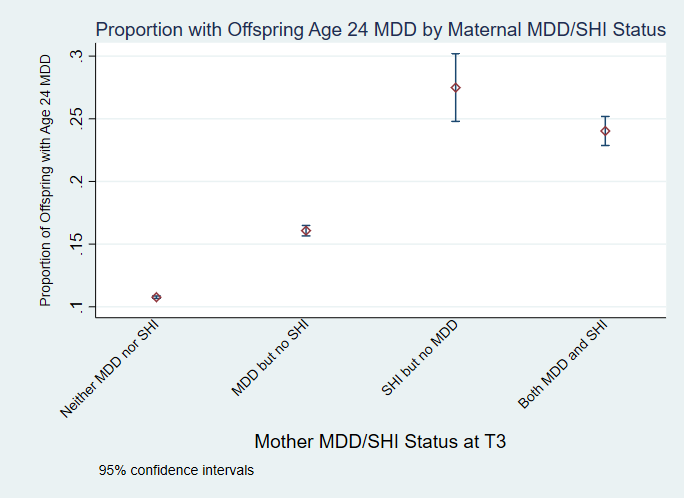

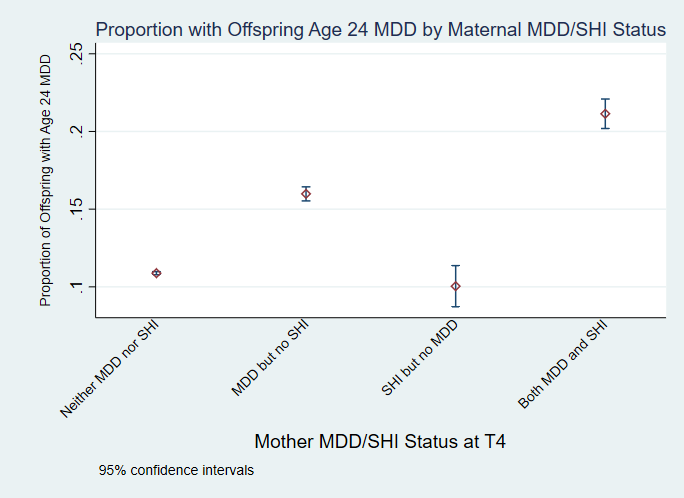

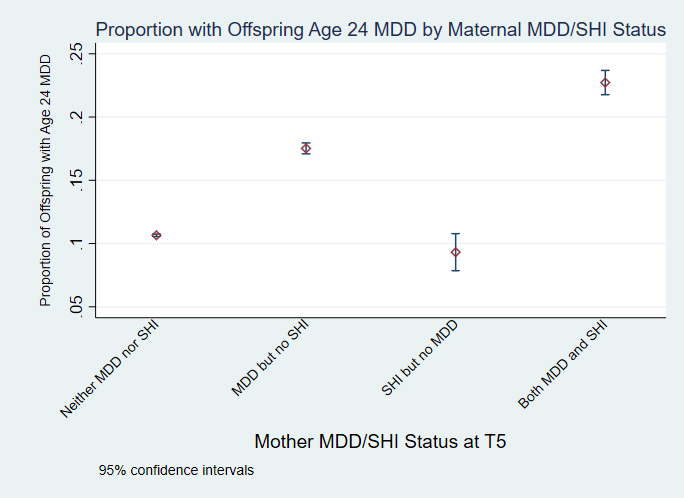

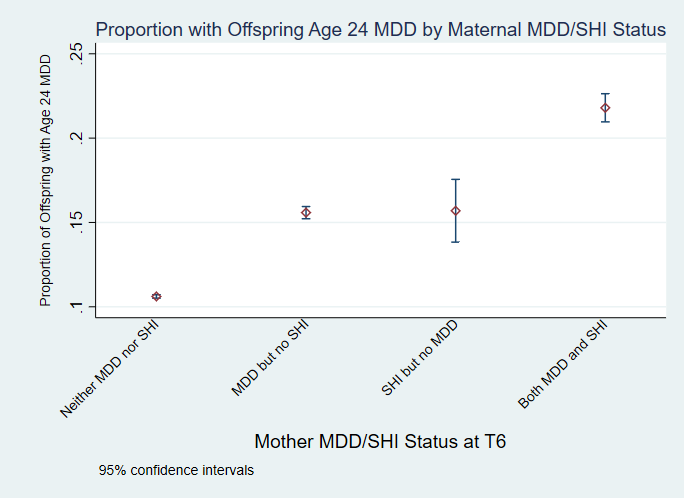

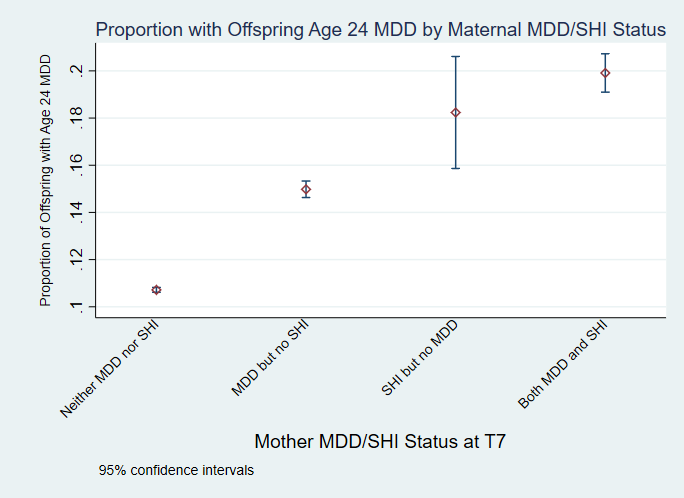

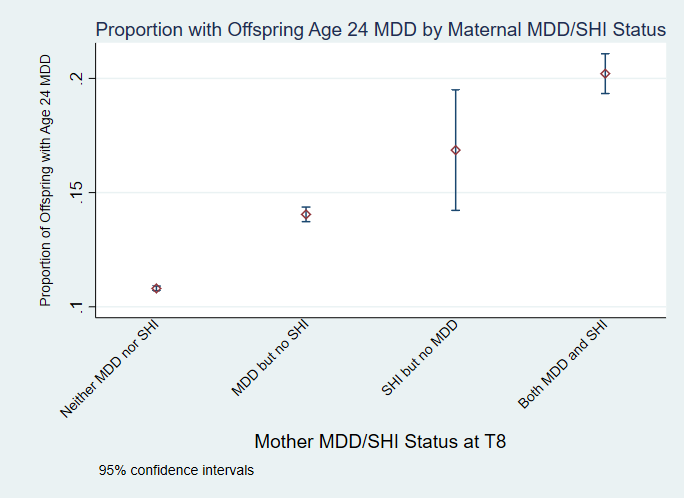

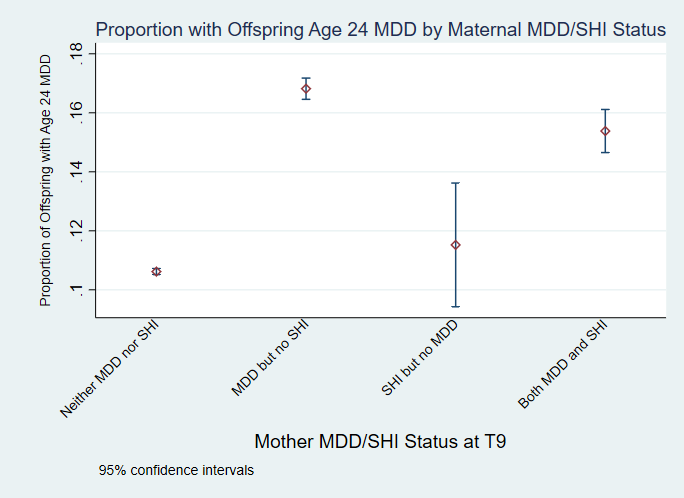

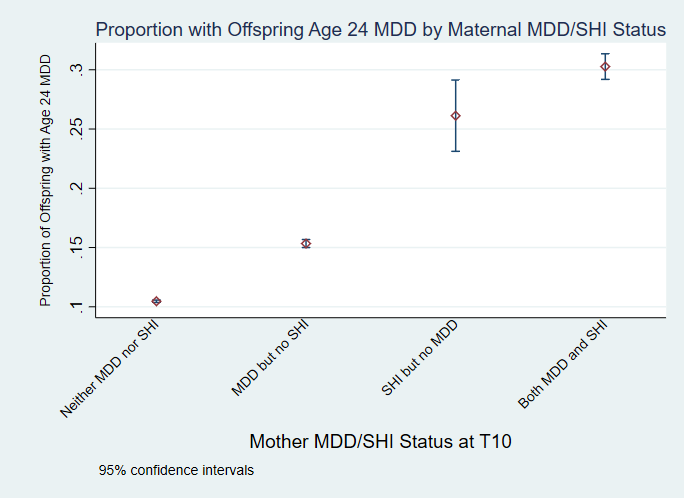

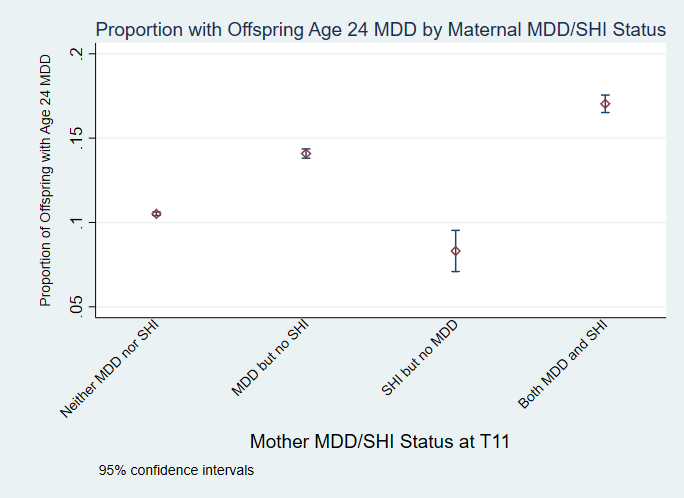
**

**eFigures 23-24. Proportion of offspring with age 24 self-harm by frequency of maternal self-harm ideation (SHI) and major depressive disorder (MDD) (*N* = 8,425).**


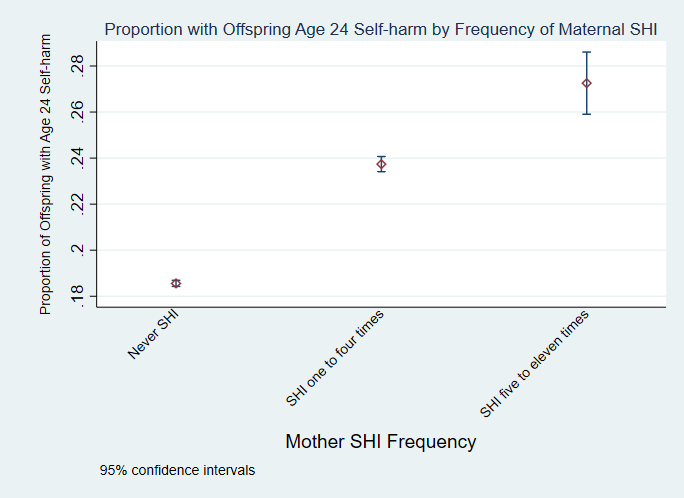

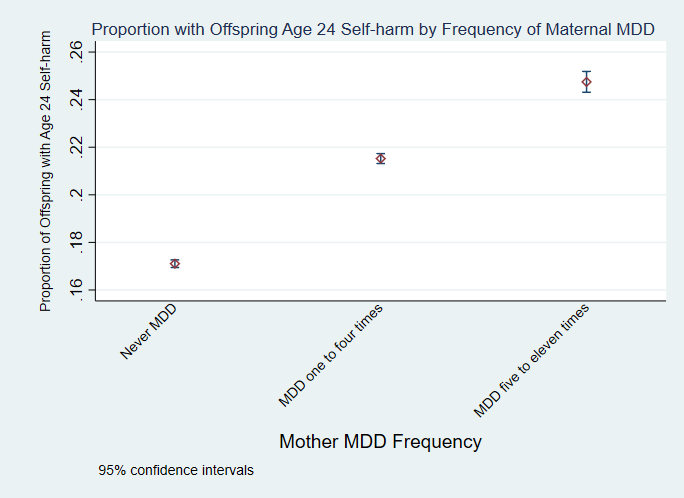


**eFigures 25-26. Proportion of offspring with age 24 major depression by frequency of maternal self-harm ideation (SHI) and major depressive disorder (MDD) (*N* = 8,425).**


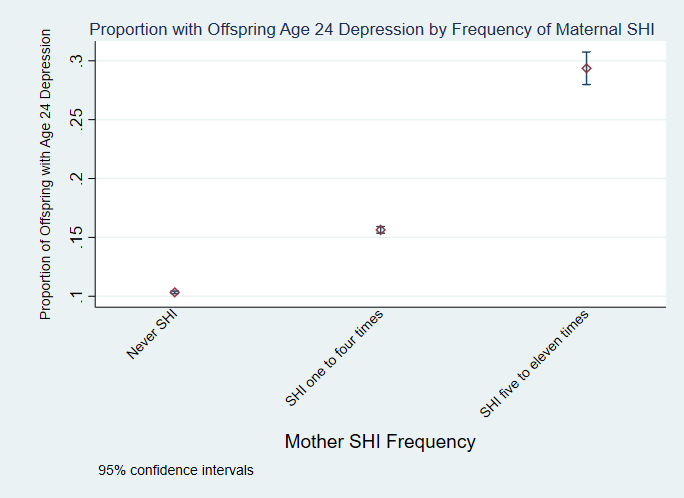

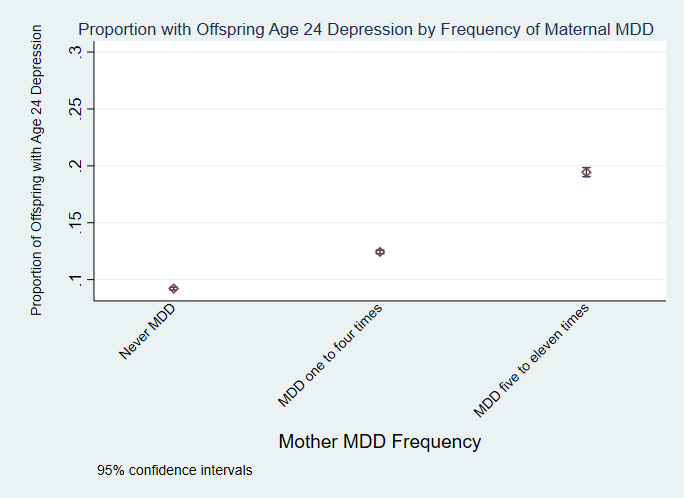

Supplement: Supplementary file 1 [file mmc1.docx]
